# Editorial Notes

**Published:** 1894-10

**Authors:** 


### Editorial Notes.

Dr. William S. Gottheil has removed to 37 West Fiftieth street, New York.

The second annual session of "Regular" Colored Physicians of the State of Georgia will meet in Macon, October 31st.

Dr. Samuel Edwin Milliken was married on October 3d, to Miss Sallie Haynes Gibbs, of Dallas, Texas. They will be at home at 36 West Fifty-ninth street, New York, after December 5th.

The twenty-fifth annual session of the Medical Society of Virginia will be held at Richmond on October 23d.

The Dixie Interstate Fair will be held at Macon, Ga., from October 23d to November 8th, 1894. There will be a better exposition of the resources of the South, and especially of the State of Georgia, than has ever been seen in one collection, and there will be a larger attendance than has ever gathered on a similar occasion.

Attractions grand and varied in character have been provided for the entertainment and amusement of the multitudes of sightseers who will visit this great Interstate Exhibition.

Maryland Clinical Society.—Stated meeting held Friday, October 5th. The 297th regular meeting of the Clinical Society was called to order by the President, Dr. J. Edwin Michael.

The following officers were elected for the ensuing year:

Dr. J. H. Branham, President; Dr. J. M. Hundley, Vice-President; Dr. H. O. Reik, Recording Secretary; Dr. William K. Robinson, Corresponding Secretary; Dr. W. J. Todd, Treasurer; Dr. Aaron Friedenwald, new member of Finance Committee; and Drs. C. Hampson Jones, Henry B. Thomas, and T. C. Gilchrist, as Executive Committee. H. O. Reik, Secretary, 525 N. Howard street.

THIS month we send to each of our subscribers, who are in arrears, a statement of their indebtedness to us. We respectfully ask that each of our friends who receive one of these notices remit the amount at once. The amount due by each is small, but the aggregate is quite a sum. We shall endeavor to give the next year the best matter that can be procured in medical circles, and we do not wish to have to drop a single one of our subscribers, and ask that the amount due us be promptly forwarded, and we will endeavor to make THE JOURNAL second to none the ensuing year.

---

MISSISSIPPI VALLEY MEDICAL ASSOCIATION.—The twentieth annual meeting of this well known organization will be held, as previously announced, at Hot Springs, Ark., November 20, 21, 22, and 23, 1894.

The general Committee of Arrangements held their final meeting on September 1st, in the city of St. Louis, there being present, in addition to the committee, President Scott of Cleveland, Vice-President Strauss of St. Louis, and Secretary Woodburn of Indianapolis. Dr. T. E. Holland, of Hot Springs, presided. All of the preliminary arrangements for the meeting of the association were completed.

Owing to the hearty co-operation of the railroads, the arrangements in this direction will surpass those of any previous meeting. On motion of Dr. I. N. Love, of St. Louis, a committee of five railroad officials was appointed to secure the desired reduction of rates. This committee consists of Messrs. Townsend, Crane, Snyder, Wishard, and Ives.

The interest and enthusiasm manifested in all parts of the country concerning the meeting in November is certainly remarkable. The fact that Hot Springs is to be the place of meeting is probably an inducement for many to attend. From the large number of favorable responses to his preliminary announcements, the Secretary feels justified, even thus early, in predicting an attendance double that of any previous meeting of the association.

Let every doctor who can possibly leave home for a few days go to Hot Springs in November. Let him take his family and his friends, and not only a profitable meeting but a royal good time will reward him for his exertions. Frederick C. Woodburn, Secretary, 399 College avenue, Indianapolis, Ind.

